# Re-elevation of T-wave from day 2 to day 4 after successful percutaneous coronary intervention predicts chronic cardiac systolic dysfunction in patients with first anterior acute myocardial infarction

**DOI:** 10.1007/s00380-012-0313-y

**Published:** 2012-12-22

**Authors:** Fumie Nishizaki, Hirofumi Tomita, Hiroaki Yokoyama, Takumi Higuma, Naoki Abe, Akiko Suzuki, Tomohide Endo, Shunta Tateyama, Yuji Ishida, Tomohiro Osanai, Ken Okumura

**Affiliations:** Department of Cardiology, Hirosaki University Graduate School of Medicine, 5 Zaifu-cho, Hirosaki, 036-8562 Japan

**Keywords:** Acute anterior ST segment elevation acute myocardial infarction, Serial electrocardiography change, Re-elevation of T-wave, Chronic cardiac function

## Abstract

This study evaluates the clinical significance of re-elevation of T-wave in patients with ST segment elevation acute myocardial infarction (STEMI) undergoing successful percutaneous coronary intervention (PCI). Resolution of ST elevation within 24 h after reperfusion is associated with better outcome. However, little is known about the serial electrocardiography (ECG) changes and their significance. Seventy-five patients (52 men; 66 ± 1 years) with the first anterior STEMI in whom 12-lead ECG was recorded every day from day 0 to day 8 after PCI were studied. JT interval was quartered (points 1–5), and the deviations from isoelectric line at each point were analyzed in leads V2, V3, and V4. Serial ECG showed ST resolution and T-wave inversion within 2 days after PCI in all patients at the middle of JT interval (point 3), and subsequent re-elevation of T-wave on day 4 in 73 patients (97.3 %). The patients were divided into two groups: Group A (*n* = 37) with less JT deviation changes (<0.25 mV) from day 2 to day 4 at point 3; and Group B (*n* = 38) with greater JT deviation changes (≥0.25 mV). Group B had less retrograde collateral flow and longer JT interval in the acute phase, and lower left ventricular ejection fraction (LVEF), worse regional contractility, and higher plasma brain natriuretic peptide levels at 6 months after the onset than Group A (all *P* < 0.05). The JT deviation change was negatively correlated with and an independent predictor for LVEF in the chronic phase. Re-elevation ≥0.25 mV of T-wave at the middle of JT interval after successful PCI predicts chronic cardiac systolic dysfunction in patients with first anterior STEMI.

**Electronic supplementary material:**

The online version of this article (doi:10.1007/s00380-012-0313-y) contains supplementary material, which is available to authorized users.

## Introduction

Previous studies suggested that early resolution of ST elevation on a 12-lead electrocardiogram (ECG) within 24 h after reperfusion therapy in patients with acute myocardial infarction (AMI) reduces the infarct size and is associated with successful myocardial reperfusion, lower mortality, and better left ventricular function [1–4]. However, these studies have largely focused on ECG changes within 24 h after the reperfusion therapy and those at just one point, such as either 60 or 80 ms after the J point. Although there have been a few studies that have analyzed serial daily ECG changes after the reperfusion therapy, not all patients in these studies underwent successful reperfusion [5–7].

In the present study, we quantitatively analyzed serial changes over JT interval on ECG during acute phase of first anterior ST segment elevation acute myocardial infarction (STEMI) treated with successful percutaneous coronary intervention (PCI), and evaluated the incidence and clinical relevance of JT interval changes.

## Patients and methods

### Study patients

Seventy-five patients with first anterior STEMI admitted to the Hirosaki University Hospital from February 2007 to October 2010, in whom 12-lead ECG was serially recorded every day through day 8 after admission, were enrolled (52 men and 23 women with a mean age of 66 (range 33–89) years) retrospectively. All patients underwent emergency coronary angiography and successful reperfusion therapy of the left anterior descending artery (LAD) by PCI with bare metal stents within 12 h after the onset (acute phase). STEMI was diagnosed by an episode of chest pain continuing over 30 min associated with ST segment elevation at the J point in two or more contiguous leads, and an increased plasma level of creatine phosphokinase-MB (CPK-MB) twice higher than the normal upper limit [8, 9]. Patients who had previous myocardial infarction evaluated by both ECG and their history, left bundle branch block, left ventricular hypertrophy, ventricular pacing, or treatment with drugs having potential effects on ECG such as antiarrhythmics were excluded. Patients in whom serial daily ECG was not recorded for any reason were also excluded. This study was approved by the ethics committee of our institution.

### Electrocardiographic evaluation

**Fig. 1:**
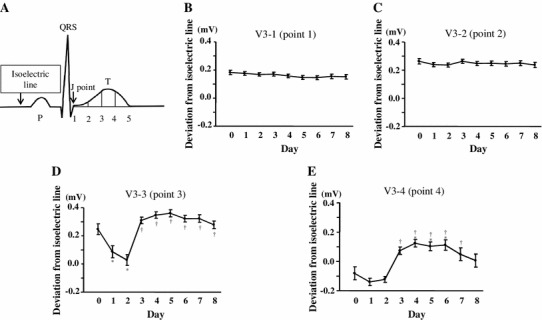
Serial changes of JT deviation. **a** Schema of ECG representing isoelectric line and quartered JT interval (points 1–5). Isoelectric line was defined as a level of the preceding TP segment. **b**–**e** JT deviation from isoelectric line at each point in lead V3 from day 0 after PCI through day 8. *P < 0.05 vs. day 0; ^†^ *P* < 0.05 vs. day 2

A 12-lead ECG was recorded on admission and every day from the day immediately following PCI (day 0) through day 8. We quartered JT interval, which starts at J point (point 1) and ends at T-wave terminal point (point 5), and evaluated the JT deviation from the isoelectric line at each point (point 1 through point 4) with hand-held calipers (Fig. 1a) in leads V2, V3, and V4 as a mean of three successive beats. Isoelectric line was defined as a level of the preceding TP segment. These measurements were performed by two cardiologists blinded to all clinical and angiographic findings. We also measured previously reported ECG markers related to left ventricular (LV) function or clinical outcomes in patients with STEMI such as sum of ST segment elevation (V2, V3, and V4) before and after PCI, ST segment elevation resolution, magnitude of negative T-wave (V2, V3, and V4) 24 h after PCI, and QRS score on admission [10–12].

### Left ventricular function and coronary artery flow

Cardiac catheterization including coronary angiography and left ventriculography was performed at approximately 14 days (subacute) and 6 months after the onset (chronic phase) as well as in the acute phase. We measured LV end-diastolic volume index (LVEDVI), LV end-systolic volume index (LVESVI), and LV ejection fraction (LVEF) by left ventriculography with the right anterior oblique view. Regional contractility score was assessed in the anterobasal, anteromedial, and apical segments according to Coronary Artery Surgery Study guidelines. Wall motion for each segment was given by a numerical code of 1 (normal), 2 (moderately hypokinetic), 3 (severely hypokinetic), 4 (akinetic), 5 (dyskinetic), or 6 (aneurysmal) [13]. Antegrade coronary blood flow was evaluated using the Thrombolysis In Myocardial Infarction (TIMI) scale [14], and all patients had a final TIMI grade of II or III in the acute phase. We further evaluated retrograde collateral flow to the LAD in the acute phase as follows: 0 (none), 1 (filling of side branches of the artery without visualization of the epicardial segment), 2 (partial filling of the epicardial segment), and 3 (complete filling of the epicardial segment) [15]. All patients, regardless of TIMI flow, received oral antiplatelet drugs including low-dose aspirin and clopidogrel or ticlopidine.

### Blood samples

Blood samples were taken from all patients, and plasma levels of creatine phosphokinase (CPK), CPK-MB, glucose, brain natriuretic peptide (BNP), as well as white blood cell count were measured in our hospital chemistry laboratory using standard techniques. BNP levels were also measured in the subacute phase and in the chronic phase. Since plasma C-reactive protein (CRP) levels were not serially measured in the study patients, they were evaluated from another group of patients.

### Statistical analysis

Data are expressed as mean ± standard error of mean (SEM). The unpaired *t* test or *χ* ^2^ test was used to compare differences between two groups. One-way analysis of variance was used for statistical analysis among multiple groups followed by the Tukey–Kramer honestly significant difference test. A linear Pearson analysis and multiple stepwise regression analysis were performed for correlation studies. Statistical analyses were performed using JMP 9 software (SAS, Cary, NC, USA). A *P* value of less than 0.05 was considered statistically significant.

## Results

### Time course of ECG changes

**Fig. 2:**
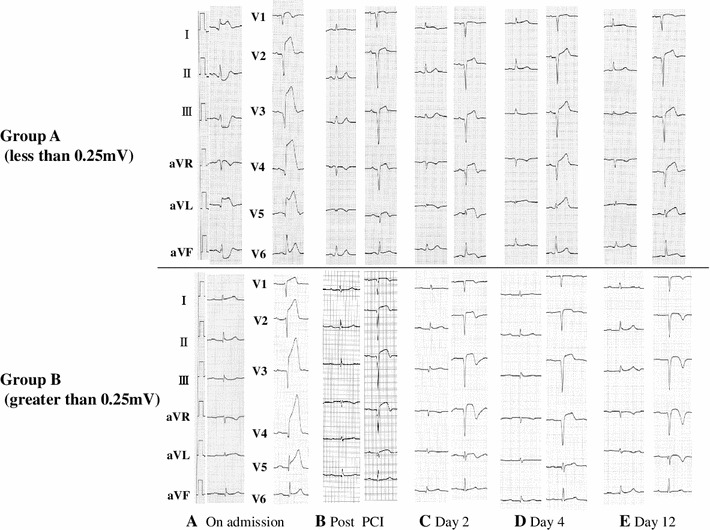
Representative serial ECG changes obtained from the patients in Groups A and B. **a** ST segment elevation was detected in leads V1–V5. **b**, **c** ST segment resolution and terminal T-wave inversion were observed within 2 days after percutaneous coronary intervention (*PCI*). **d** On day 4, re-elevation of the T-wave was detected in leads V2–V5. **e** On day 12, terminal T-wave inversion was detected again

Representative serial ECG changes are shown in Fig. 2. All patients showed ST resolution and T-wave inversion within 2 days after PCI (Fig. 2b, c). Of all 75 patients, 73 (97.3 %) showed re-elevation of T-wave within 4 days after PCI (Fig. 2d). They all showed T-wave inversion again at approximately 2 weeks after the onset (Fig. 2e).

Detailed analyses on serial changes in the JT deviation from the isoelectric line at each point in lead V3 are shown in Fig. 1b–e. No daily changes were found at point 1 (V3-1) (Fig. 1b). Although a slightly higher JT deviation was found at point 2 (V3-2) compared with point 1 (V3-1), again there were no daily changes of the JT deviation at point 2 (V3-2) (Fig. 1c). Most striking changes were found at point 3 (V3-3), where the JT deviation was significantly diminished in the first 2 days, and reached the level of the isoelectric line (Fig. 1d). Surprisingly, the JT deviation on day 3 was markedly elevated again, even more so than on day 0, and continued to hold the elevated JT deviation for several days. Almost the same daily changes as in the V3-3 were observed at point 4 (V3-4) (Fig. 1e). These changes of the JT deviation at points 3 and 4 are likely to reflect T-wave inversion and subsequent re-elevation of the T-wave. Indeed, the timing of point 3 on day 2 and day 4 was before the peak of T-wave in 84 %–93 % of the patients, whereas that of point 4 was after the peak in 74 %–85 % of the patients (Tables 1, 2). The same analyses were performed in leads V2 and V4, and observations similar to those for V3-3 were made (Supplemental Figures 1 and 2). The inter- and intra-observer coefficients of variation for ECG analyses were 11.7 % ± 2.1 % and 7.3 % ± 1.8 %, respectively.

**Table 1:** Timing of points 3 and 4 relative to the T-wave peak (trough) at day 2

		Point 3	Point 4
V2	Before T-wave peak	62 (84 %)	11 (15 %)
	After T-wave peak	12 (16 %)	63 (85 %)
V3	Before T-wave peak	63 (85 %)	19 (26 %)
	After T-wave peak	11 (15 %)	55 (74 %)
V4	Before T-wave peak	63 (85 %)	16 (22 %)
	After T-wave peak	11 (15 %)	58 (78 %)

**Table 2:** Timing of points 3 and 4 relative to the T-wave peak (trough) at day 4

		Point 3	Point 4
V2	Before T-wave peak	69 (93 %)	12 (16 %)
	After T-wave peak	5 (7 %)	62 (84 %)
V3	Before T-wave peak	64 (86 %)	9 (12 %)
	After T-wave peak	10 (14 %)	65 (88 %)
V4	Before T-wave peak	69 (93 %)	16 (22 %)
	After T-wave peak	5 (7 %)	58 (78 %)

### Relationships between JT deviation changes and baseline characteristics

To assess the clinical significance of T-wave inversion and subsequent re-elevation of the T-wave, we evaluated relationships between JT deviation changes and the clinical course of the patients. For this purpose, we calculated the JT deviation changes between day 2, showing nadir in JT deviation, and day 4, showing re-elevated JT deviation at point 3 in leads V2, V3, and V4 (V2-3, V3-3, and V4-3) (Fig. 1d). We averaged these three values, and defined a mean deviation change (mDev) in each patient. The patients were divided into two groups based on the median value of mDev (0.25 mV): Group A with mDev <0.25 mV (*n* = 37) and Group B with mDev ≥0.25 mV (*n* = 38). Detailed analyses in each group regarding serial daily changes of the JT deviation at each point in leads V2, V3, and V4 are shown in Supplemental Figures 3–5.

There were no significant differences in the baseline characteristics including sex, age, body mass index, coronary risk factors, and reperfusion time from onset between the two groups (Table 3). Furthermore, the severity of myocardial infarction assessed by maximum CPK value, maximum CPK-MB value, Killip classification and Forrester subset, number of stenotic vessels, and percentage of final TIMI grade III did not differ between the two groups. White blood cell count and plasma glucose level on admission, incidence of preinfarction angina, initial TIMI flow grade, and culprit lesion also had no significant differences between the two groups. Regarding ECG markers related to clinical outcomes, sum of ST segment elevation before and after PCI, ST segment elevation resolution, and QRS score ≥5 on admission did not differ between the two groups. However, magnitude of negative T-wave 24 h after PCI was significantly lower in Group A than in Group B (*P* < 0.05). Furthermore, better retrograde collateral flow to the LAD in the acute phase was found in Group A in comparison with Group B (*P* < 0.05). Drug therapy such as angiotensin I-converting enzyme inhibitors or angiotensin II type 1 receptor blockers, β-blockers, and statins in the patients at discharge and after 6 months was not different between the two groups, nor was target-lesion revascularization after 6 months (Supplemental Table 1).

**Table 3:** Baseline characteristics of the patients

	Group A (*n* = 37)	Group B (*n* = 38)	*P* value
Men	28 (75 %)	24 (63 %)	0.24
Mean age (years)	65 ± 2	67 ± 2	0.53
Body mass index (kg/m^2^)	25.5 ± 0.66	24.9 ± 0.76	0.53
History of smoking	20 (54 %)	18 (47 %)	0.56
Hypertension	27 (73 %)	30 (78 %)	0.55
Diabetes mellitus	17 (46 %)	18 (47 %)	0.90
Dyslipidemia	20 (54 %)	24 (63 %)	0.42
Atrial fibrillation	3 (8 %)	2 (5 %)	0.62
CRBBB	2 (5 %)	6 (16 %)	0.15
STE before PCI (mV)	1.4 ± 0.2	1.7 ± 0.2	0.20
STE after PCI (mV)	0.7 ± 0.1	0.7 ± 0.1	0.83
STE resolution (mV)	0.7 ± 0.1	1.1 ± 0.1	0.12
QRS score on admission ≥5	11 (26 %)	9 (47 %)	0.07
Magnitude of negative T-wave (mV)	0.20 ± 0.03	0.31 ± 0.04	0.02
Time from onset to reperfusion (min)	307 ± 24	304 ± 21	0.91
Maximal CPK (IU/l)	5001 ± 749	4748 ± 452	0.77
Maximal CPK-MB (IU/l)	322 ± 38	422 ± 40	0.07
White blood cell count (×10^3^/mm^3^)	12.0 ± 0.6	11.0 ± 0.5	0.20
Blood glucose level (mg/dl)	165 ± 11	170 ± 68	0.78
Preinfarction angina	20 (54 %)	14 (36 %)	0.13
Initial TIMI flow 0 or 1	29 (78 %)	33 (87 %)	0.33
Culprit lesion (segment 6)	21 (57 %)	17 (45 %)	0.30
Number of stenotic vessels			
1	23 (62 %)	26 (68 %)	
2	11 (30 %)	9 (24 %)	0.83
3	3 (8 %)	3 (8 %)	
Killip classification (A: *n* = 36)			
I	29 (80 %)	31 (76 %)	
II	1 (3 %)	5 (13 %)	0.20
III/IV	6 (2 %)	2 (5 %)	
Intra-aortic balloon pump	19 (51 %)	22 (58 %)	0.57
Final TIMI grade III	32 (86 %)	29 (76 %)	0.26
Forrester subset (A: *n* = 37, B: *n* = 35)			
I	16 (43 %)	16 (46 %)	
II	5 (14 %)	1 (3 %)	0.37
III/IV	16 (43 %)	18 (51 %)	
Collateral flow (Rentrop’s grade)			
0	11 (30 %)	23 (61 %)	
1	18 (49 %)	9 (24 %)	0.02
2	8 (22 %)	6 (16 %)	

Atrial fibrillation includes persistent and paroxysmal atrial fibrillation

*CRBBB* complete right bundle branch block, *CPK* creatine phosphokinase, *STE* sum of ST segment elevation, *TIMI* Thrombolysis In Myocardial Infarction

To investigate whether pericarditis accompanying STEMI contributes to the re-elevation of the T-wave, we evaluated the time course of plasma CRP levels from the onset (day 0) through day 8 in another group of six patients with first anterior STEMI admitted to our hospital. Although similar JT deviation changes to that shown in Fig. 1 was observed in all patients, no re-elevation of plasma CRP levels was observed during this period.

### JT interval between the two groups

**Fig. 3:**
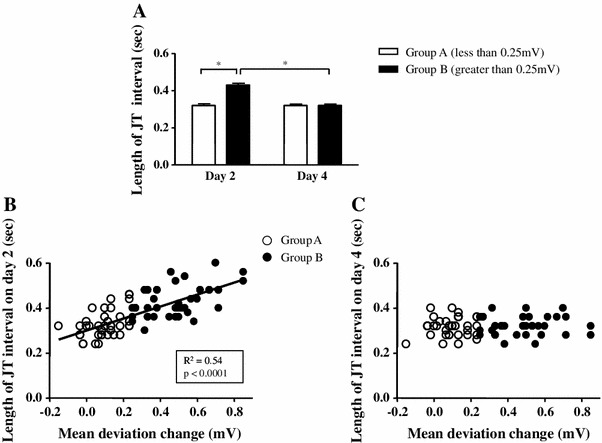
Length of JT interval and its relationships with mean JT deviation changes (mDev) on days 2 and 4. **a** Comparisons of length of JT interval between the two groups on days 2 and 4. Relationships between length of JT interval and mDev on day 2 (**b**) and day 4 (**c**). The mDev in Group A is less than 0.25 mV at the middle of the JT interval (point 3), while that in Group B is equal or greater than 0.25 mV. *P < 0.05

As each point where we measured JT deviation was determined by quartering the JT interval, it is possible that length of JT interval could affect the value of JT deviation. Therefore, we investigated the relationships between the length of the JT interval and JT deviation in lead V3. The JT interval in Group B on day 2 was significantly longer than that in Group A (0.43 ± 0.01 vs. 0.32 ± 0.01 ms, *P* < 0.05), but was not different on day 4 (0.32 ± 0.01 vs. 0.32 ± 0.01 ms, *P* = 0.64) (Fig. 3a). There was a significant positive correlation between the length of JT interval and the mDev on day 2 (*R* ^2^ = 0.54, *P* < 0.0001, Fig. 3b), but no correlation on day 4 (*R* ^2^ = 0.006, *P* = 0.52) (Fig. 3c). These findings indicate that the longer JT interval in Group B on day 2 was associated with the greater value of mDev. Similar findings were obtained when we used the QT interval instead of the JT interval (Supplemental Figure 6). The same analyses were performed using JTc and QTc intervals, adjusted by heart rate, and almost the same results as JT and QT intervals were obtained.

### Comparison of cardiac function between the two groups

**Fig. 4:**
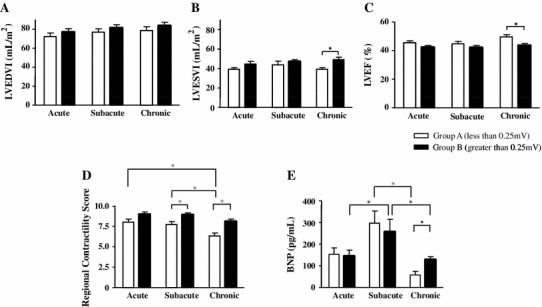
Comparisons of cardiac function between Groups A and B in each phase. Comparisons of left ventricular end-diastolic volume index (*LVEDVI*) (**a**), left ventricular end-systolic volume index (*LVESVI*) (**b**), left ventricular ejection fraction (*LVEF*) (**c**), regional contractility score (**d**), and plasma brain natriuretic peptide (*BNP*) levels (**e**) between the two groups in the acute phase, in the subacute phase, and in the chronic phase. *P < 0.05

We compared the LV function between the two groups in the acute, subacute (2 weeks after the onset), and chronic (6 months after the onset) phases. LVEDVI between the two groups was similar in all phases (Fig. 4a). By contrast, LVESVI in Group B was greater than that in Group A in the chronic phase (49.2 ± 2.4 vs. 39.3 ± 1.7 ml/m^2^, *P* < 0.05), whereas it was not different in the acute and subacute phases (Fig. 4b). Consistent with this, LVEF in Group B was lower than that in Group A in the chronic phase (43.6 % ± 1.3 % vs. 49.6 % ± 1.5 %, *P* < 0.05) (Fig. 4c). Although regional contractility score showed no difference between the two groups in the acute phase, the score was significantly lower in Group A than in Group B in the subacute (7.7 ± 0.4 vs. 8.9 ± 0.2, *P* < 0.05) and chronic phases (6.3 ± 0.4 vs. 8.1 ± 0.3, *P* < 0.05) (Fig. 4d). Plasma BNP levels were not different between the two groups in the acute and subacute phases, although they were relatively high in the subacute phase compared to those in the acute phase. Importantly, the plasma BNP levels in Group B in the chronic phase were more than twice as high as those in Group A (128.1 ± 16.6 vs. 57.5 ± 11.5 pg/ml, *P* < 0.05) (Fig. 4e).

### Relationships between mean JT deviation change and cardiac function

**Fig. 5:**
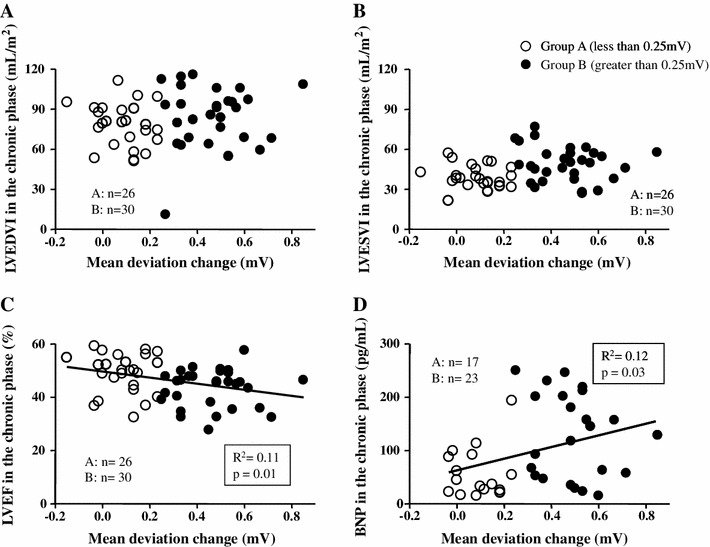
Concomitantly with JT deviation change (mDev) and cardiac function or plasma BNP (*BNP*) levels. Correlation studies between mDev and left ventricular end-diastolic volume index (*LVEDVI*) (**a**), left ventricular end-systolic volume index (*LVESVI*) (**b**), left ventricular ejection fraction (*LVEF*) (**c**), or plasma BNP levels (**d**)

To investigate whether the mDev in the acute phase predicts cardiac function in the chronic phase, correlation studies were performed. The mDev was not significantly correlated with LVEDVI (*R* ^2^ = 0.01, *P* = 0.47) (Fig. 5a) and correlated weakly with LVESVI (*R* ^2^ = 0.06, *P* = 0.06) (Fig. 5b) in the chronic phase. However, it was negatively correlated with LVEF (*R* ^2^ = 0.11, *P* = 0.01) (Fig. 5c) and positively correlated with plasma BNP levels (*R* ^2^ = 0.12, *P* = 0.03) (Fig. 5d) in the chronic phase. There was no correlation between the mDev and maximum CPK-MB levels (*R* ^2^ = 0.03, *P* = 0.14). Furthermore, a multiple stepwise regression analysis was performed for the LVEF in the chronic phase (dependent variable) with age, sex, body mass index, presence of coronary risk factors, maximum CPK-MB levels, mDev, magnitude of negative T-wave, and QRS score ≥5 on admission as independent variables. The analysis showed that the mDev and maximum CPK-MB levels were independent contributors to the LVEF in the chronic phase after adjusting for age and sex (Table 4).

**Table 4:** Multivariate regression analysis for left ventricular ejection fraction in the chronic phase

	Coefficient (*β*)	SEM	*P* value
Mean JT deviation change	−0.254	0.620	0.030
Dyslipidemia	−0.213	0.139	0.060
Maximum CPK-MB	−0.501	0.001	<0.001

*CPK-MB* creatine phosphokinase-MB

The sensitivity, specificity, positive and negative predictive values, and predictive accuracy of re-elevation >0.25 mV of T-wave at the middle of JT interval from day 2 to day 4 after PCI for the prediction of chronic LVEF less than 40 % were 69 %, 51 %, 30 %, 85 %, and 55 %, respectively.

## Discussion

In the present study, we assessed serial daily changes in 12-lead ECG in patients with first anterior STEMI successfully treated with PCI, and found re-elevation of the T-wave from day 2 to day 4 after the PCI in leads V2, V3, and V4 in almost all patients. Moreover, we demonstrated that in Group B, patients with a greater mDev (≥0.25 mV) at the middle of the JT interval (point 3) in leads V2, V3, and V4 in the acute phase had lower LVEF and higher plasma BNP levels than patients in Group A (<0.25 mV) at 6 months after the onset. The mDev value was also one of the independent negative contributors to LVEF in the chronic phase. These findings indicate that re-elevation of the T-wave at the middle of the JT interval from day 2 to day 4 after successful PCI can be a simple predictive clinical marker for cardiac systolic dysfunction at 6 months after the onset of AMI.

Previous studies have focused on the ST segment changes in the early stage within 24 h after reperfusion therapy [4, 12, 16–18]. However, there have been only a few studies that have analyzed serial daily JT interval changes after the onset of AMI. Shimizu et al. [6] evaluated ST segment changes at 60 ms after the J point during 1 week after the onset of anterior AMI, and showed that patients with elevated ST segment changes had increased LVESVI and decreased LVEF at approximately 2.5 months after the onset. Although these findings are partly consistent with our results, their study was done in the era of thrombolysis therapy, and approximately 50 % of the patients had an unsuccessful reperfusion (total or subtotal occlusion immediately after thrombolysis). More recently, Cortadellas et al. [7] showed that ST segment elevation at 80 ms after the J point at 72 h after admission correlated negatively with LVEF and positively with LV dilatation at 1 year after the onset. However, coronary angiography was not performed in the first 72 h in their study; therefore, it was unclear whether all patients had a successful reperfusion. Thus, in these two previous studies the influence of incomplete reperfusion of the culprit lesion to the LV function in the chronic phase could not be excluded. In the present study, we confirmed that all patients had a successful reperfusion by PCI with final TIMI grade III in 81 % and grade II in 19 %. We also evaluated JT deviation not just at one point such as either 60 or 80 ms after the J point, but at quartered points of the JT interval (points 1–4). Thus, our methods for evaluation of deviation on ECG involve not only the degree of JT deviation but length of JT interval after reperfusion therapy. The longer JT interval in Group B on day 2 was likely to affect the locations of points 3 and 4, which were shifted to the inverted T-wave site, and thereby a decreased JT deviation from the isoelectric line was observed on day 2.

The mechanism by which Group B patients showed lower LVEF in the chronic phase than those in Group A is less obvious, and cannot be fully elucidated from the present study. It may be related to the differences in the regional contractility score and retrograde collateral flow to the LAD between Groups A and B. The patients in Group A had a better retrograde collateral flow than those in Group B in the acute phase. The regional contractility score in Group A was significantly improved in the subacute and chronic phases compared with that in the acute phase, whereas no improvement in Group B was observed (Fig. 4d). Better retrograde collateral flow in the acute phase could partly contribute to the improved regional contractility score and a better regional function in Group A in the subacute and chronic phases. Indeed, it has been reported that collateral-derived flow maintains myocardial viability and results in better regional function in the chronic phase, even in patients with severe regional dysfunction in the acute phase [19].

It should be noted that regional pericarditis might be one of the potential causes of re-elevation of the T-wave. The diagnosis of regional pericarditis following AMI is difficult to make, but it should be considered in patients with recurrent chest pain in the setting of atypical T-wave evolution and persistent ST segment elevation [20]. In this study, only three patients (4 %) had recurrent chest pain without ECG changes during 8 days after PCI. Furthermore, no friction rub was detected. We also investigated the time course of plasma CRP levels in another group of patients with first anterior STEMI. No patient had re-elevation of CRP, especially between days 2 and 4, while all showed JT deviation change similar to that of the study patients. Thus the effects of pericarditis on JT deviation change were subtle, although its possibility could not be completely excluded.

Infarct expansion and extension may explain re-elevation of T-wave. Infarct expansion is characterized by dilatation and thinning of the area of infarction, and is not explained by additional myocardial necrosis [21]. Infarct extension, on the other hand, is characterized by additional myocardial necrosis and is manifested by recurrent chest pain, ST segment re-elevation, and rise in serum CPK level. In this study, no patient showed recurrent chest pain and re-elevation of serum CPK concomitantly with JT deviation change. Thus re-elevation of T-wave may not be caused by extension, but may be explained by expansion of myocardial infarction.

## Clinical implications

We provided evidence that the LV function in the chronic phase can be predicted by recording ECG every day from day 2 to day 4 after onset of first anterior STEMI, but relatively low diagnostic accuracies suggest the possible involvement of other factors such as maximum CPK-MB levels. Since patients with greater JT deviation change (≥0.25 mV) from day 2 to day 4 had poor LV function in the chronic phase, intensive treatment may be required to prevent LV remodeling in such patients.

## Limitations

There are some limitations in this study. First, generalization of our results would be limited by the small number of study patients and single-center retrospective analysis. Second, our findings can be applied only to the patients with anterior myocardial infarction. ECG analyses in patients with myocardial infarction in the other regions are of interest. Finally, plasma CRP levels were not serially measured in the study patients. Despite these limitations, our evaluation of JT deviation changes from day 2 to day 4 in leads V2, V3, and V4 by quartering the JT interval, particularly at the middle of the JT interval (point 3), appears to be a useful method to predict cardiac systolic function in the chronic phase. Further large-scale clinical studies are warranted.

## Conclusion

We provide evidence that biphasic change in the T-wave in leads V2, V3, and V4 commonly occur after successful PCI in patients with first anterior STEMI. Since the degree of re-elevation of the T-wave was correlated with LV functions and was an independent predictor of LVEF in the chronic phase, ECG changes in the acute phase can be a clinically useful prognostic factor for acute myocardial infarction.

## Electronic supplementary material

Below is the link to the electronic supplementary material.

Supplementary material 1 (PDF 215 kb)
