# Supplementary material for: Re-elevation of T-wave from day 2 to day 4 after successful percutaneous coronary intervention predicts chronic cardiac systolic dysfunction in patients with first anterior acute myocardial infarction
Source: Heart Vessels. 2012 Dec 22;28(6):704–13. doi: 10.1007/s00380-012-0313-y (PMC3830194; doi:10.1007/s00380-012-0313-y)
Supplement: Supplementary file 1 — Supplementary material 1 (PDF 215 kb) [file 380_2012_313_MOESM1_ESM.pdf]

Supplementary Material (Heart and Vessels)

Re-elevation of T-wave from day 2 to day 4 after successful percutaneous coronary intervention predicts chronic cardiac systolic dysfunction in patients with first anterior acute myocardial infarction

Fumie Nishizaki, Hirofumi Tomita, Hiroaki Yokoyama, Takumi Higuma, Naoki Abe, Akiko Suzuki, Tomohide Endo, Shunta Tateyama, Yuji Ishida, Tomohiro Osanai, and Ken Okumura

From the Department of Cardiology, Hirosaki University Graduate School of Medicine, Hirosaki, Japan

Correspondence to: Ken Okumura, MD

Department of Cardiology, Hirosaki University Graduate School of Medicine,

5 Zaifu-cho, Hirosaki, 036-8562 Japan

Tel: 81-172-39-5057 Fax: 81-172-35-9190

E-mail: okumura@cc.hirosaki-u.ac.jp

Supplemental figure 1

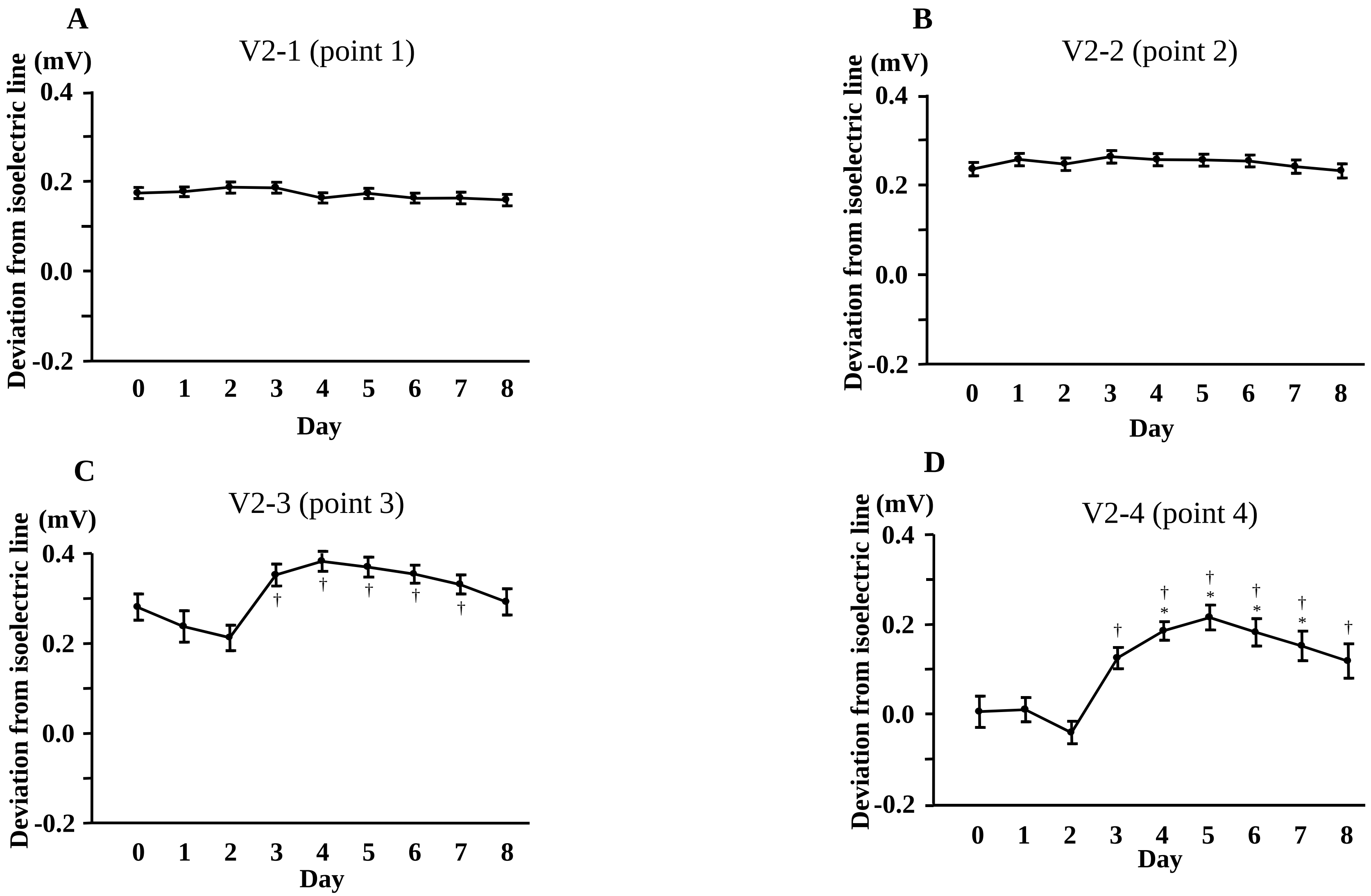

Supplemental figure 1. Chronological changes of JT deviation from isoelectric line at each point (point 1 to point 4) in lead V2. * $p<0.05$ versus day 0, † $p<0.05$ versus day 2.

Supplemental figure 2

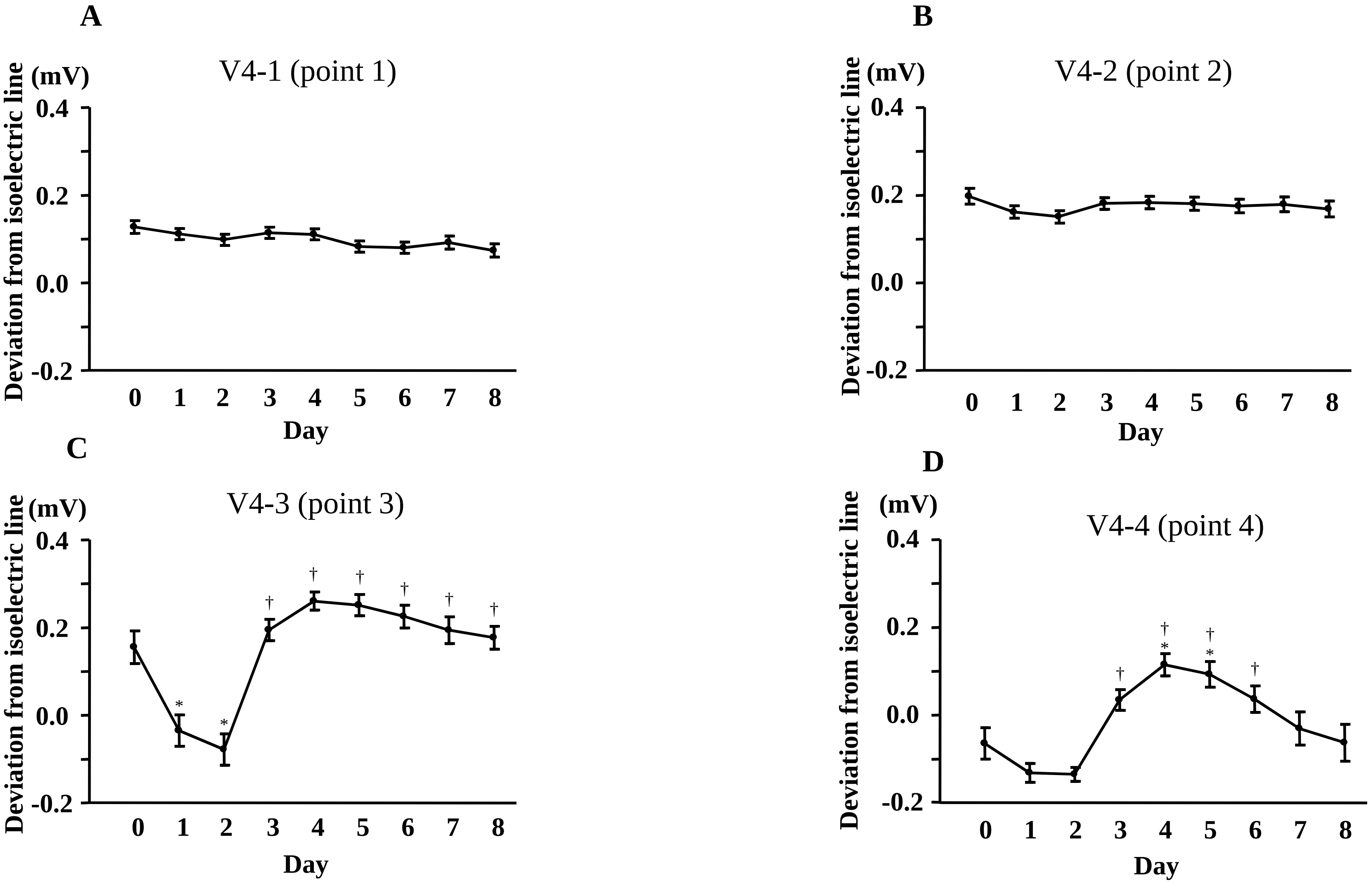

Supplemental figure 2. Chronological changes of JT deviation from isoelectric line at each point (point 1 to point 4) in lead V3. * $p<0.05$ versus day 0, † $p<0.05$ versus day 2.

Supplemental figure 3

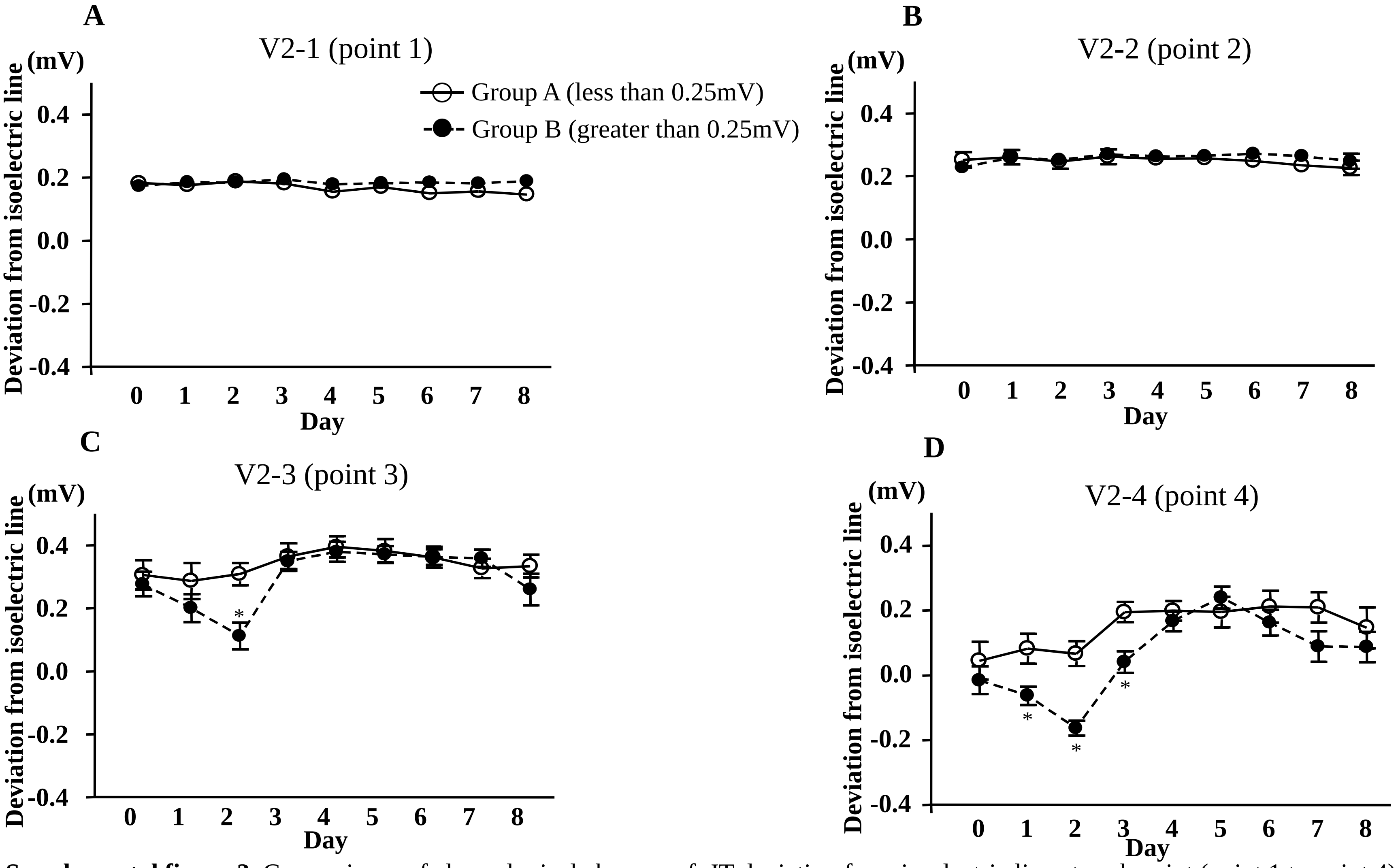

Supplemental figure 3. Comparisons of chronological changes of JT deviation from isoelectric line at each point (point 1 to point 4) in lead V2 between the Group A and Group B. The Group B had lower JT deviation on day 2 in point 3 (V2-3)(**C**) and on day 1-3 in point 4 (V2-4)(**D**) than the Group A. * $p<0.05$ between the Group A and the Group B.

Supplemental figure 4

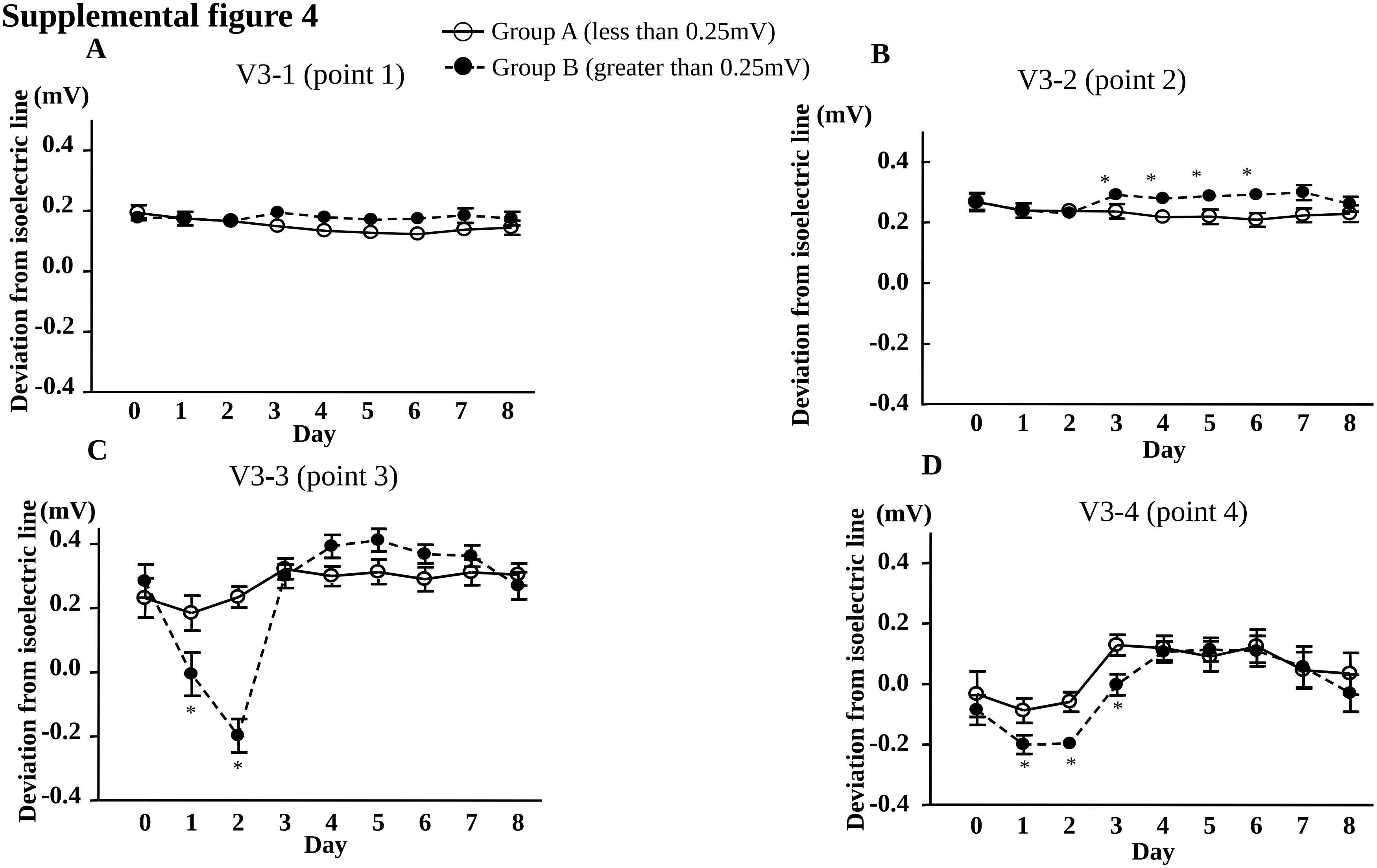

Supplemental figure 4. Comparisons of chronological changes of JT deviation from isoelectric line at each point (point 1 to point 4) in lead V3 between the Group A and Group B. The Group B had lower JT deviation on day 1 and day 2 in point 3 (V3-3)(**C**) and on day 1-3 in point 4 (V3-4)(**D**) than the Group A. * $p<0.05$ between the Group A and the Group B.

Supplemental figure 5

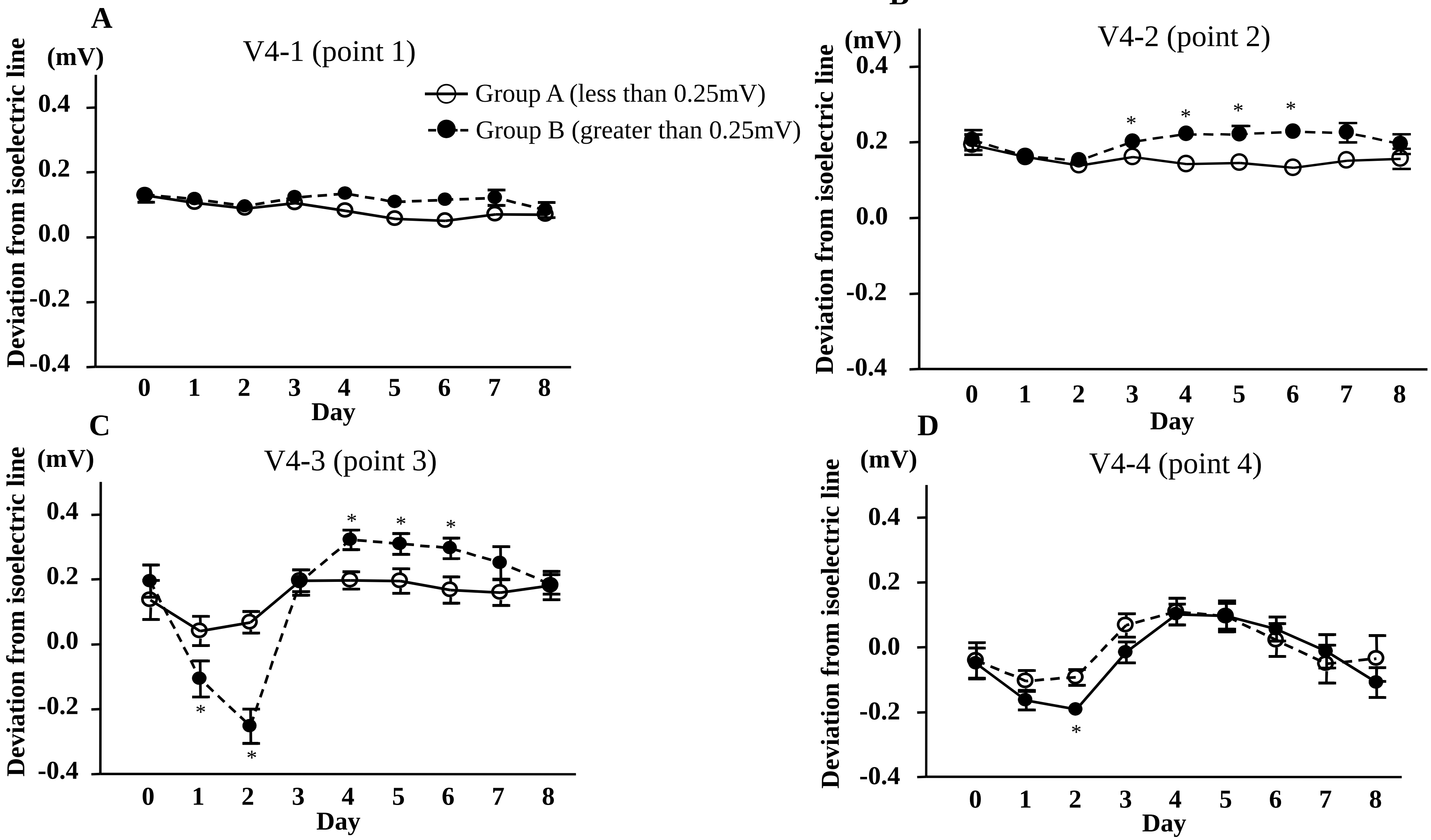

Supplemental figure 5. Comparisons of chronological changes of JT deviation from isoelectric line at each point (point 1 to point 4) in lead V4 between the Group A and Group B. The Group B had lower JT deviation on day 1and day 2 in point 3 (V4-3)(**C**) and on day 2 in point 4 (V4-4)(**D**) than the Group A. * $p<0.05$ between the Group A and the Group B.

Supplemental figure 6

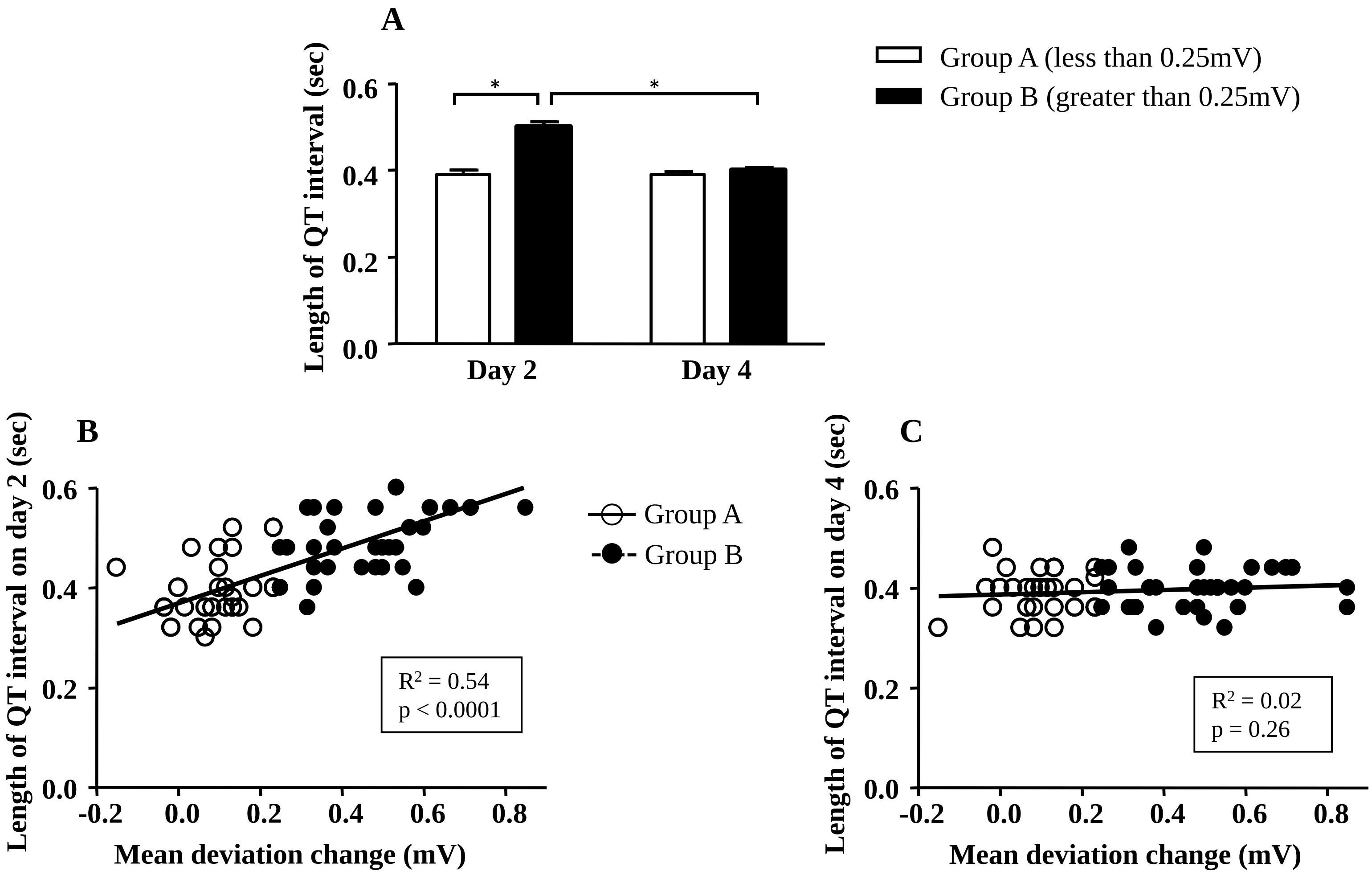

Supplemental figure 6. The length of QT interval and its relationships with mean deviation changes at day 2 and day 4. **A**. Comparisons of length of QT interval between the two groups on day 2 and on day 4. **B and C**. Relationships between length of QT interval and mean deviation changes on day 2 (**B**) and on day 4 (**C**). The Group A has mean deviation changes from day 2 to day 4 averaged in leads V2-V4 less than 0.25 mV at the middle of JT interval (point 3), while the Group B has greater than 0.25 mV. *p<0.05.

Supplemental table 1 **Drug therapy and restenosis of culprit lesion**

	Group A (n=37) (Less than 0.25mV)	Group B (n=38) (Greater than 0.25mV)	p value
At discharge (A:n=36, B:n=38)			
ACE inhibitor or ARB	33 (92%)	38 (100%)	0.07
β blocker	33 (92%)	33 (87%)	0.50
Statin	24 (65%)	24 (63%)	0.75
After six months (chronic phase) (A:n=26, B:n=28)			
ACE inhibitor or ARB	25 (96%)	27 (96%)	0.96
β blocker	24 (92%)	27 (96%)	0.51
Statin	22 (85%)	23 (82%)	0.81
TLR of culprit lesion in the chronic phase (A:n=27, B:n=31)	9 (33%)	9 (29%)	0.72

ACE indicates angiotensin I-converting enzyme, ARB; angiotensin II type 1 receptor blocker, TLR; target lesion revascularization.
